# Why have total cholesterol levels declined in most developed countries?

**DOI:** 10.1186/1471-2458-11-641

**Published:** 2011-08-11

**Authors:** Simon Capewell, Earl S Ford

**Affiliations:** 1 University of Liverpool, Liverpool, United Kingdom; 2 Centers for Disease Control and Prevention, Atlanta, GA, USA

## Abstract

**Background:**

Our paper addresses three major public health issues: cholesterol, statins and policies to prevent cardiovascular disease.

**Discussion:**

Total cholesterol levels in whole populations have fallen substantially in the USA, UK and most other developed countries. This has greatly contributed to decreases in cardiovascular disease deaths. The evidence identifying diet as the major contributor to these historical falls in cholesterol is powerful and consistent. Large falls occurred before statins were introduced. Additional substantial falls occurred before statins were widely used.

Now, up to 14% adults in Western populations currently receive statins for primary prevention. Furthermore, because diet is now only slowly improving, the statin contribution currently appears proportionately larger.

**Summary:**

In conclusion, diet change explains most of the historical falls in cholesterol. Until very recently, the contribution from statins has been surprisingly modest. Furthermore, many middle income countries may have neither the resources nor the infrastructure for mass statin therapy.

Further substantial falls in cholesterol are therefore unlikely to be obtained simply by increased use of statins or dietary advice to individuals if unsupported by the wider environment. This further emphasises the need for more effective structural policies. Regulatory and fiscal interventions could easily eradicate industrial transfats, halve the intake of dietary saturated fat, and subsidise healthier fats.

## Background

In recent decades, mean population total cholesterol levels have fallen by as much as 1.0 mmol/l (40 mg/dl) in most developed countries. Understanding why is crucial for planning future health strategies to prevent cardiovascular disease (CVD). Cholesterol has major public health importance as a powerful cause of atherosclerosis and thrombosis, hence coronary heart disease and ischemic stroke. Every 1% fall in mean population total cholesterol levels decreases CVD mortality by approximately 2.5% [1]. Thus, recent population cholesterol falls explain up to 25% of the concomitant decreases in cardiovascular mortality in the USA, Canada and elsewhere [2]. However, cholesterol levels in every country remain substantially above the optimal level of approximately 4.5 mmol/l [1-3].

Total blood cholesterol consists of sub-fractions. Approximately two thirds being low density lipoprotein (LDL, the main villain for CVD), some 30% comes from high density lipoprotein, (HDL, which is protective against CVD) and the remainder comes from very low density lipoproteins (triglycerides) [1]. Although trend data on LDL and HDL are limited outside the USA, happily, changes in total cholesterol levels are highly correlated with LDL [1].

We now address a key question: *Why have total cholesterol and LDL levels fallen in most developed countries? Is it mainly statins, or diet or other factors? *

## Discussion

First licensed in the late 1980s, statin prescribing first increased slowly, then exponentially from the late 1990s to now reach approximately 14% of US and UK adults [3]. It is therefore perhaps useful to distinguish three separate time-periods, the "pre-statin", "early statin" and "current statin" eras.

For the **"pre-statin" era**, the substantial population falls in cholesterol must obviously be attributed to other factors, principally diet. Between 1960 and 1994, US cholesterol levels decreased by approximately 0.46 mmol/l (18 mg/dl). Equally large falls were also seen internationally, including Finland and New Zealand (Table 1).

**Table 1:** Cholesterol trends in whole populations in the "Pre-statin", "Early statin" and "Current statin" eras*

Population	Survey Years	Data Source (approximate sample size)	Number of Adults on statins	Adults on long term statins (Primary and secondary prevention)	Mean total cholesterol reduction in whole population Men *(women)* mmol/l	Estimated contribution from statins† (%)	First author & reference
***"The Pre-Statin Era"***							
**USA**	1960-1994	NHANES (44,980)	0	0	-0.41 *(-0.52 )*	0%	Carroll 2005 [14]
**New** Zealand	1982-1993	ARCOS (3,800)	0	0	-0.45 *(-0.66 )*	0%	Capewell 2000 [15]
**Finland**	1982-1997	FinRisk (13,000)	0	0	-0.64 *(-0.66 )*	0%	Laatikainen 2005 [16]
**Scotland**	1985-1994	MONICA (5,900)	0	0	-0.10 *(-0.40 )*	0%	Capewell 1999 [17]
***"The Early Statin Era"***							
**England**	1981-2000	BRHS & HSE (17,500)	280,000	< 1%	-0.14 *(-0.07)*	5%	Unal 2004 [18]
**Ireland**	1985-2000	Kilkenny & SLAN surveys (7,900)	60,000	2%	-0.35 *(-0.22)*	5%	Bennett 2006 [19]
**Italy**	1980-2000	RIFLE and OEC surveys (62,000)	900,000	3%	-0.33 *(-0.37 )*	6%	Palmieri 2009 [20]
**Sweden**	1986-2002	MONICA & INTERGENE (8,800)	280,000	4%	-0.55 *(-0.68)*	6%	Bjorck 2009 [21]
**Iceland**	1981-2006	IHA population studies (17,700)	4,000	2%	-0.80 *(-0.95)*		Aspelund 2010 [22]
***"The Current Statin Era"***							
**UK**	2003-2006	HSE (15,500)	4.4 million	11%	-0.20 *(- 0.20)*	41%	Mindell 2009 [23]
**USA**	1994-2002	NHANES (14,530)	18 million	9%	-0.078 *(-0.08)*	88%	Carroll 2005 [14]
**USA**	1999-2006	NHANES (18,050)	28 million	14%	-0.106 *(-0.11)*	95%	Ford 2009 [3]

TABLE FOOTNOTE

*Statin eras defined pragmatically as: "Pre-statin era": before 1997; "Early-statin era": 1997 - 1999; and "Current statin era": since 2000, using the most recent year for which data are available.

†Methodology for calculating statin contribution is detailed in Additional File 1, Appendix B.

Statin uptake in Sweden increased substantially between 2002 and 2009 (Eriksson M. et. al. J Intern Med. 2011;269:219-231).

Abbreviations: ARCOS = Auckland Region Coronary Or Stroke; BRHS = British Regional Heart Study; HSE = Health Survey for England; MONICA = Monitoring Trends and Determinants on Cardiovascular Diseases; NHANES = National Health and Nutrition Examination Survey; OEC = Osservatorio Epidemiologico Cardiovascolare; RIFLE = Risk Factors and Life Expectancy; SLAN = Survey of Lifestyle, Attitudes, and Nutrition.

In the **"early statin era" **(the late 1990s), individuals clearly benefited from medication. Initially, the emphasis was on secondary prevention for selected patients with recognised disease. Yet, even after publication of Air Force/Texas Coronary Atherosclerosis Prevention Study in 1998, statins were initially prescribed to only a small fraction of eligible adults (consistently less than 4%). Population effects would clearly have been limited. Yet substantial total cholesterol falls of 0.14-0.8 mmol/l (5-30 mg/dl) were observed in diverse populations (Table 1).

**In the "current statin era" **prescribing rates are now substantial, ranging from 9% in Sweden [4] to 12% in the UK and 14% in the USA [3]. Furthermore, because recent absolute cholesterol falls have been modest, statins may now explain perhaps 45% of the falls in the UK between 2003 and 2006, and almost all (88% - 98%) of the recent fall in the US population between 1999 and 2006 (Table 1).

### Future strategy options for population-wide cholesterol reduction

Further cholesterol reduction will be essential to reduce the future burden of CVD. The key challenge is to identify the most effective and cost-effective strategy.

### Future Strategy Option 1: PHARMACOLOGY

#### Identify and treat more individuals with statins?

Statins powerfully lower cholesterol in individuals, yet simple logic suggests that statin benefits across an entire population may be much diluted. Doubling the current level of statin prescribing would increase costs but achieve surprisingly modest reductions in cardiovascular mortality [[5] and Additional File 1, Appendix A].

Firstly because of the law of diminishing returns: expanding the criteria for statin prescribing would capture individuals with progressively lower absolute event rates. Secondly, because control of hypercholesterolaemia (defined as the proportion of all hypercholesterolaemic individuals subsequently achieving blood levels below 5 mmol/l (200 mg/dl) remains frustratingly poor, typically ranging from 17% to just 31% [3]. This mediocre population impact probably reflects progressive attrition down Tugwell's "staircase", whereby effectiveness decreases at each step in the complex clinical pathway from initial awareness, access, coverage, screening, targeting, diagnosis, and compliance through to patient adherence [5]. Even after successful testing and advice, only a proportion of patients repeatedly fill a statin prescription [6]. *Statin compliance (adherence) *is problematic, approximately half of patients actually take their statin medication every day [6]. And *long-term persistence *is also poor: 12 months after commencing therapy, barely 50% continue on statins [5]. Moreover, *statin dosing *is often suboptimal, with a correspondingly weaker cholesterol reduction [5,6]. Thus *statin effectiveness *in ordinary clinical practice will clearly be decreased, perhaps reducing cholesterol by approximately 1 mmol/l (40 mg/dl), rather than the 2 mmol/l (80 mg/dl) efficacy achieved in selected patients in controlled trials [7].

### Diet and Cholesterol

The extensive evidence identifying dietary fats as the major contributor to cholesterol levels ranges from randomised controlled trials to population-wide natural experiments. "Bad" saturated fats and trans fats substantially increase LDL and total cholesterol levels. Conversely, "good" polyunsaturates and monounsaturates raise HDL and decrease LDL and total blood cholesterol levels. Thus replacement is beneficial in the laboratory, and in the community [6]. For instance, in Poland in 1990, communist subsidies for animal fats disappeared and a free market provided relatively cheap vegetable oils, fruit and vegetables. The polyunsaturate/saturate ratio improved substantially, and coronary death rates fell by 24% within five years. Corresponding trends were reported in East Germany, Hungary and the Czech Republic [7].

Less powerful factors modestly reducing LDL and total cholesterol levels should also be briefly considered. Most are nutritional. **Plant-based diets **(fresh fruit, vegetables and wholegrain cereals) and **Mediterranean type diets **significantly decrease total cholesterol and LDL [8]. Furthermore, CVD mortality is approximately 25% lower in individuals routinely consuming a vegetarian or "prudent diet" (lots of fruit and vegetables, nuts, cereals and fish, little meat or dairy) and is correspondingly higher with a "Western diet" (more meat and dairy) [8]. Changes in diet and other lifestyle behaviors are powerfully influenced by socio-economic factors. Affluent and educated groups generally display healthier dietary patterns, these being facilitated by greater disposable income. Statin use is likewise generally greater in more affluent groups [9-12].

Modest cholesterol reductions might also be attributed to **hormone replacement therapy**. However, after the adverse publicity surrounding the Women's Health Initiative results, prescribing almost halved and, furthermore, any cholesterol lowering benefits would be mainly confined to peri-menopausal women. Sustained **exercise **may decrease total cholesterol in individuals by approximately 0.10 mmol/l (4 mg/dl). Optimistically assuming a 5% increase in US or UK population prevalence of moderate physical activity since 1980 might therefore mean, at best, a 0.005 mmol/l (0.2 mg/dl) reduction in total cholesterol [2].

Conversely, increasing obesity trends will adverse affect cholesterol levels. However, the effect is surprisingly small - every unit increase in **body mass index **may increase non-HDL cholesterol by only about 0 045 mmol/l (2 mg/dl). Furthermore, equally substantial cholesterol falls have actually been observed in all sections of the population, whether obese, overweight or normal weight [13].

### Future Strategy Option 2: *PUBLIC HEALTH POLICIES TO PROMOTE HEALTHIER DIETS?*

Large cholesterol decreases occurred before the widespread use of statins. The evidence consistently identifies dietary fat changes as the main contributor. However, recent diet improvements in the US and UK have been frustratingly slow. Effective diet policy interventions do exist - legislative, fiscal and social levers have worked elsewhere [4,8-10]. These levers could be used in the US, UK and elsewhere to eliminate industrial transfats, halve the intake of dietary saturated fat and increase the consumption of polyunsaturates and monounsaturates.

## Summary

Cholesterol reduction represents a major public health priority in the campaign to prevent cardiovascular disease. Statins are generally viewed as the only solution; but this ignores both the historical facts and the scientific evidence.

Total cholesterol levels in whole populations have fallen substantially in the USA, UK and most other developed countries. Large falls actually occurred before statins were introduced [14-17]. Further substantial falls occurred before statins were widely used [18-23]. Moreover, the evidence identifying diet as the major contributor to these historical falls in cholesterol is powerful and consistent.

Now, however, things are less simple. Up to 14% adults in Western populations currently receive statins for primary prevention. And because diet is now only slowly improving, the statin contribution currently appears proportionately larger. Yet, many middle income and low income countries may have neither the resources nor the infrastructure for mass statin therapy. Further substantial falls in cholesterol are unlikely to be obtained simply by increased use of statins, nor by further dietary advice to individuals without additional support from the wider socio-economic environment.

This highlights the need to consider more effective structural policies. The evidence underpinning regulatory and fiscal interventions is impressive. Such population-wide policies could easily eradicate industrial transfats, halve the intake of dietary saturated fat, and subsidise healthier fats.

## Competing interests

SC was Vice-Chair of the UK NICE Programme Development Group on Cardiovascular Disease Prevention in Populations. Dr Ford has nothing to declare.

## Authors' contributions

SC wrote the first draft. ESF then substantially extended and enhanced it. Both were involved in subsequent drafts and finalisation of the paper.

## Pre-publication history

The pre-publication history for this paper can be accessed here:

http://www.biomedcentral.com/1471-2458/11/641/prepub

## Supplementary Material

Additional file 1 **Appendix A**. The potential mortality benefits if statins were prescribed to a larger proportion of hypercholesterolaemic adults. ***Appendix B***. Methodology: estimating the statin contribution to the overall reduction in population cholesterol levels. Click here for file
