# Supplementary material for: Why have total cholesterol levels declined in most developed countries?
Source: BMC Public Health. 2011 Aug 11;11:641. doi: 10.1186/1471-2458-11-641 (PMC3199603; doi:10.1186/1471-2458-11-641)
Supplement: Additional file 1 — Appendix A. The potential mortality benefits if statins were prescribed to a larger proportion of hypercholesterolaemic adults. Appendix B. Methodology: estimating the statin contribution to the overall reduction in population cholesterol levels. [file 1471-2458-11-641-S1.DOC]

***APPENDICES***

***Appendix A.*** The potential mortality benefits if statins were prescribed to a larger proportion of hypercholesterolaemic adults

***Appendix B.*** Methodology: estimating the statin contribution to the overall reduction in population cholesterol levels.

***Appendix A:* The potential mortality benefits if statins were prescribed to a larger proportion of hypercholesterolaemic adults**

| **Country**  [Adult Population] | **Year** | **Source of population data** | **% of adult population on long term statins for primary prevention** | | **Annual mortality reduction** | **Annual mortality reduction *IF* statins were prescribed to 80% of eligible adults** | **Mortality gain** | **Study** |
| --- | --- | --- | --- | --- | --- | --- | --- | --- |
| **USA**  [177 million] | 2000 | NHANES | | 8% | 16,580 | 35,890 | **19,310** fewer deaths | Capewell 2009 ^A^ |
|  |  |  | |  |  |  |  |  |
| **England & Wales**  [35 million] | 2000 | HSE | | 3% | 145 | 3405 | **3,260** fewer deaths | Capewell 2006 ^B^ |
|  |  |  | |  |  |  |  |  |
| **Ireland**  [4 million] | 2000 | SLAN | | 6% | 45 | 610 | **565** fewer deaths | Kabir 2006 ^C(1)^ |

^A^Capewell S, O'Flaherty M, Ford ES, Critchley JA. Am J Cardiol 2009, 103:1703-9.

^B^Capewell S, Unal B, Critchley JA, McMurray JJ. Heart 2006, 92:521-3.

^C^Kabir Z, Bennett K, Shelley E, Unal B, Critchley J, Feely J, et al. QJM 2006, 99:523-30.

*HSE = Health Survey for England; NHANES = National Health and Nutrition Examination Survey;*

*SLAN = Survey of Lifestyle, Attitudes, and Nutrition.*

***Appendix B* Methodology: estimating the statin contribution to the overall reduction in population cholesterol levels**

The approximate proportion of the recent reductions in population total cholesterol attribute little to current statin prescribing can be crudely estimated. (Table 1). For example, between 1994 and 2002, total cholesterol fell 0.078 mmol/l among approximately 200 million US adults aged 20 years and over (14). In 2002, approximately 18.25 million adults were receiving statins (3).

The proportion of the cholesterol drop attributable to statins can therefore be calculated as:

Numbers of Americans receiving statins in 2002 * anticipated fall in cholesterol from statin trials * suboptimal dosing adjustment * imperfect daily compliance adjustment /

fall in mean population total cholesterol levels between 1994 and 2002.

= 18.25 million adults X 2 mmol/l decrease X 0.75 X 0.5

0.078 mmol/l fall in 200 million adults

= 13,800,000

15,600,000

= 88 %

This 88% contribution from statins increases to approximately 98% when considering the US population between 1999 and 2006. Similarly, statins apparently explained approximately 45% in the UK 2003-2006 (Table 1). `

We acknowledge the uncertainty in such a crude estimate. However, the assumptions about compliance and uptake appear reasonable, because if they were any less severe, the final contribution would produce a value exceeding 100%, which is clearly implausible.

Reference List

(1) Kabir Z, Bennett K, Shelley E, Unal B, Critchley J, Feely J, et al. The population mortality benefits of maximizing the number of eligible patients receiving appropriate cardiology treatments in Ireland. QJM 2006 Aug;99(8):523-30.
